# Psychotically driven aggression is associated with greater mentalizing challenges in psychotic spectrum disorders

**DOI:** 10.1186/s12888-020-02868-7

**Published:** 2020-09-29

**Authors:** Anette GM Johansson, Malin Källman, Lennart Högman, Marianne Kristiansson, Håkan Fischer, Sven Bölte

**Affiliations:** 1 Department of Clinical Neuroscience, Karolinska Institutet, 171 77 Stockholm, Sweden; 2 Center of Neurodevelopmental Disorders (KIND), Centre for Psychiatry Research; Department of Women’s and Children’s Health, Karolinska Institutet &, Stockholm Health Care Services, Region Stockholm, 113 30 Stockholm, Sweden; 3 Centre for Psychiatry Research, 113 64 Stockholm, Sweden; 4 Department of Psychology, Stockholm University, 106 91 Stockholm, Sweden; 5 Department Neurobiology, Care Sciences and Society, Karolinska Institutet, Stockholm, Sweden; 6 Child and Adolescent Psychiatry, Stockholm Health Care Services, Region Stockholm, Stockholm, 118 61 Sweden; 7 Curtin Autism Research Group, School of Occupational Therapy, Social Work and Speech Pathology, Curtin University, Perth, Western Australia Australia

**Keywords:** Violence, Psychosis, Schizophrenia, Aggression, Social cognition, DMASC-MC, Theory of mind

## Abstract

**Background:**

Some aggressive acts committed by individuals with psychotic spectrum disorders (PSD) are understandable in the context of interpersonal conflict or goal attainment, yet others are unpredictable, arising from delusions or hallucinations (psychotically driven aggressive acts, PDA). It is unknown if there are underlying differences in cognitive or perceptive social cognition in relation to aggression motivation in PSD.

**Method:**

We compared differences in social cognition performance between 49 individuals with PSD who had committed PDA with those exhibiting other types of aggression (*n* = 31) (non-PDA) and to community controls (*n* = 81) on the Swedish version of Double Movie for the Assessment of Social Cognition – Multiple Choice (DMASC-MC). Participants with PSD had more than 3 months of clinical stability and substance use abstention and stable antipsychotic medication doses. General intellectual ability was assessed with the information and matrix reasoning subtests of the Wechsler Intelligence Scales.

**Results:**

The PSD group with a history of PDA exhibited lower total and perceptive social cognition scores on the DMASC-MC than the non-PDA group and controls. In addition, they also showed lower cognitive scores compared to typical controls. Lower total scores were associated with lower scores on Wechsler intelligence subtests information and matrix reasoning. Taking this into account, the PDA group still had lower social cognition scores. There were no associations of antipsychotic medication dosages, positive or negative symptoms with social cognition scores. Higher antipsychotic dosage at the time of DMASC-MC testing and social cognition scores predicted a past history of PDA.

**Conclusions:**

We conclude that impaired social cognition, particularly perceptive social cognition, is associated with PDA in individuals with PSD.

## Background

Social cognition refers to our capacity to process stimuli relevant to the understanding of other persons and their interactions. Mental processes relevant for the understanding of others’ emotions, motives, mental states and how these impinge upon oneself include social motivation, emotion recognition, social attention, and social learning [1]. It includes the ability to attribute mental states and intentions to oneself and others, an ability often referred to as cognitive empathy or theory of mind. It relies on perceiving and comprehending emotions as portrayed, or not portrayed, by others, as well as inferring thoughts and motivations according to the situation. Social cognition skills allow one to navigate in the social world, and are a prerequisite for success in relationships, at work and in other societal arenas. Social cognitive impairments vary between different neurodevelopmental and psychiatric disorders [2, 3], with both genetic and and psychosocial factors contributing to their emergence [4].

Social cognition has been thoroughly investigated in schizophrenia, see for example [5–7], and has been found to be impaired in the majority of those diagnosed with the condition. Indeed, social cognition impairments have been postulated to be an endophenotype [8] of schizophrenia given that both first degree relatives and individuals in first psychotic episodes show social cognition impairments [9]. Non-emotional cognition, such as reasoning ability and capacity for metacognition, is a significant contributor to impaired perceptive and cognitive social cognition capacities [7, 10, 11] even in individuals in the normative intelligence range. Additionally, social cognition ability has, at least in individuals with schizophrenia, been found in meta-analysis to correlate with psychosocial functioning and employability [12], indicating the importance of being able to correctly understand others communications and intentions for societal integration.

Difficulties in decoding other’s intentions and feelings generate misinterpretations, which may fuel frustration and aggression. Studies on social cognition abilities in aggressive individuals have focused on antisocial personality disorder (ASPD) [13], and psychopathic traits [14, 15]. Findings show that psychopathic traits are negatively associated with perceptive social cognition skills but not with cognitive social cognition, reflecting a possible dissociation of these two social cognition pathways [16]. While only few studies have examined social cognition in individuals with schizophrenia with a history of aggression [14, 17, 18], these suggest that aggression per se is coupled with impaired social cognition. However, not all studies have found that social cognition is more impaired in those with schizophrenia who have been aggressive compared with those who have not [19], possibly related to the recognition that aggression in those with schizophrenia broadly separates into that which begins early with a history of conduct disorder and impulsivity and that which has a later debut which is often regarded to be more intimately connected with psychotic content [20]. Yet clinically it is well known that even young persons with schizophrenia may have an early debut into violence which is driven by their psychotic experiences and which occurs without a history of conduct disorder. By examining age of debut rather than the motivation behind the aggression one may miss important information in elucidating the etiological factors of aggressive behaviours in those suffering from schizophrenia. No studies, to our knowledge, have examined social cognition in individuals with schizophrenia as a function of whether the aggression was directly driven by psychotic experiences such as delusions or hallucinations (Psychotically driven aggression – PDA) or not (non-PDA). The distinction is important to make, as this delineation of causality in aggression determines in some legal jurisdictions which care/legal pathway is chosen when somebody is charged and/or found guilty of aggressive acts.

A shortcoming of social cognition studies thus far published on psychotic spectrum disorders (PSD, here defined as schizophrenia, delusional disorder, schizoaffective disorders, as well as psychotic bipolar disorder with illness related cognitive deficits and schizotypal disorder) is that they have not seemingly controlled for possible confounding effects of semantic understanding of emotion words. Confusion over the meaning of emotion words can influence the attribution of emotion to other persons and thereby affect the interpretation of the social interplay, potentially leading to aggression.

In view of the above, we sought to study the relationship between social cognition abilities and PDA in PSD, considering the role of intellectual ability and semantic understanding of emotion words. Our hypotheses were that: (i) social cognition – both cognitive and perceptive would be more impaired in PSD individuals exhibiting PDA than those without PDA, who would be intermediate to community controls, (ii) that cognitive measures and semantic understanding would exert discrete effects on social cognition.

## Methods

### Participants

Eighty-one individuals with PSD were recruited from the Stockholm Forensic Psychiatric Project (SFPP), which was established to investigate potential links between epidemiologically based risk factors for aggression in those with PSD with psychological measures and biological parameters. Further information regarding the overarching SFPP is described elsewhere in detail [21]. This sub-study of the SFPP is a cross-sectional cohort study of persons sentenced to receive inpatient compulsory forensic psychiatric care because of aggressive acts or a history of aggressive acts. Inclusion diagnoses were PSD defined as schizophrenia, schizoaffective disorder, psychotic bipolar disorder with cognitive impairments, delusional disorder, schizotypal disorder, and/or autism spectrum disorder with psychotic episodes. All bar 2 of the individuals with autism spectrum disorder met criteria for schizophrenia. The inclusion of autism spectrum disorders with psychotic episodes and those with cognitive impairments and psychotic symptoms of bipolar disorder was made on the basis of phenotypic similarities to schizophrenia spectrum disorders in terms of social cognition impairments [5, 22] In order to be representative of the population of PSD, individuals who commit aggressive acts, comorbid substance abuse (verified remission for > 3 months before testing), attention deficit hyperactivity disorder, personality disorder and mild intellectual disability (previously assessed intelligence Quotients of 50 to 70) were included. Committed offences included threatening behaviour, assault, grievous bodily harm and manslaughter/murder. Other offences included property theft, robbery, arson or deliberate fire setting, but in these cases prior interpersonal aggression had always been observed, warranting their inclusion in the research project. Participants were aged between 20 and 60 years at enrolment and recruited between 2015 and 2019. Exclusion criteria were neurological disorders, brain damage prior to diagnosis with psychosis, untreated endocrine disorders, moderate intellectual disability and acute psychiatric illness episode. Individuals were recruited and tested in a period of mental state stability where they had had stable medication dosages for more than 3 months.

The community control group was composed of 32 individuals recruited for SFPP and 49 recruited as part of the psychometric evaluation of the Double Movie of Assessment of Social Cognition-Multiple Choice (DMASC-MC) [23] at the Centre of Neurodevelopmental Disorders at Karolinska Institutet (KIND) in Stockholm during April and May 2014. Subjects recruited as part of SFPP were selected as follows: the State Resident Address Registry at the Swedish Tax Agency was contacted and given sex and year of birth to match the sex and age of recruited PSD participants. Three controls matching each PSD participant were requested living in selected postcode areas for each randomized computerized search. The Tax agency provided the designated researcher with a list of names and addresses, who then sent out letters of invitation to these individuals, asking them to contact the research team if they were interested in participating in the research. Interested individuals completed a short telephone interview designed to exclude heredity for bipolar or psychotic disorders, ongoing substance abuse or medical conditions that met exclusion criteria. At this stage, *n* = 6 had to be excluded, owing to heredity for studied psychiatric disorders or ongoing substance use disorders. The remainder were booked for research project participation. These individuals were provided economic recompense for loss of income. The above process was repeated a number of times in order to obtain sufficient numbers of participants. Study procedure were identical to those for the PSD patients in this study. Subjects from the validation study of DMASC-MC were recruited via PFM Research in Sweden AB (www.pfmresearch.se), a company with many years of experience in doing population representative selections of research subjects for state and county authorities as well as other organizations. PFM selected 324 individuals out of their panel containing 75,000 individuals aged 13 years and older to be contacted for the DMASC-MC standardization. The sample was designed to be representative for the Swedish population in terms of sex, age and geographical location. Economic compensation was paid in form of a gift to charity or a voucher to a range of shops. Individuals agreeing to participate in the actual study received an email with a link to log in to an internet site where participants gave some background information before performing the DMASC-MC online as an internet version programmed in a combination of JavaScript and PHP and the results written to a SQL-database. Maximum answer time for each question was set at 40 s, the same as for the PSD participants.

Of these 324 individuals from the DMASC-MC normative standardization data set, 49 were selected for the present study to match the PSD participants for age and sex, still ensuring that the total average DMASC-MC scores were comparable to those of the population control group. Given a lack of older participants in the normative group, those aged over 40 years were matched one on one with SFPP individuals who did not already have a control from the SFPP sample. For younger participants where multiple participants were available for each proband the person closest to total mean population scores for DMASC-MC was selected. Along with the 32 individuals recruited via SFPP this yielded 81 individuals.

### Psychiatric assessments

PSD participants were diagnosed using case record review supplemented by semi-structured interviews. Symptoms of psychiatric disorders were rated by experienced psychiatric clinicians according to the psychotic and affective sections of World Health Organisations Schedule for Clinical Assessment in Neuropsychiatry (SCAN 2.1) [24] and diagnoses were made according to DSM-5 criteria for schizophrenia, schizoaffective disorder, delusional disorder, schizotypal disorder, and bipolar disorder with psychotic features. Current medication was rated from case records; doses of antipsychotic medication converted according to Andreasen’s model to haloperidol equivalents per day [25].

Actual psychotic symptoms were rated according to the Scale for the Assessment of Positive Symptoms (SAPS) [26], and the Scale for the Assessment of Negative Symptoms (SANS) [27]. Controls enrolled within SFPP all were interviewed using the SCAN 2.1 and symptom ratings used the above instruments. Controls in the validation study of DMASC-MC had not been asked about their mental health.

### Aggression ratings

The number and types of aggressive acts were analysed according to Cornell’s rating guide [28] based on i) contemporaneous crime investigation reports from police for crimes the person has been sentenced for, ii) reports in the forensic psychiatric care assessment about previous crimes the person has been sentenced for, iii) case records of observable aggressive incidents in hospital services prior to forensic psychiatric care where intention and psychotic experiences related to the act were recorded in close proximity to the incident, and iv) self-reports. Aggressive acts were further divided into those that were judged to be a direct result of hallucinations or delusions (PDA) and other types of aggression (instrumental or reactive interpersonal) (non-PDA), which, however, virtually all occurred during a psychotic episode. Computerized case records for each person from all psychiatric service providers in the Stockholm area since 2007 were accessed. In essence, the ratings were based on contemporaneous written accounts based on what the individual who had just committed the aggressive acts reported and observations of witnesses to the aggression. Accounts came from police interviews with the suspect and witnesses, technical evidence including photographs of injuries and crime scenes as well as information from forensic psychiatric examiners who had access to case notes from examining medical doctors in remand centres or inpatient settings where the person had been aggressive. Additionally, contemporaneous inpatient accounts of aggressive incidents were used, where both witness observations and patient accounts formed the basis for ratings. Persons who had exhibited both types of aggression were sorted into the PDA group given that this form of aggression had consistently been conducted more recently and had led to the forensic psychiatric care.

### Social cognition, emotion word comprehension and cognitive abilities

To examine their level of emotion word comprehension, PSD participants and 32 controls were asked to identify the correct synonym for 14 emotion words. There was a choice of three words for each emotion (e.g., for anger – pride, rage, despair; for fear – dread, anger and pleasure).

Social cognition skills were assessed with the 15-min-long DMASC-MC. The movie-based MASC-MC portrays the story of two young adult females (Elin and Jenny) and two young adult males (Hannes and Daniel) planning and then meeting for dinner. The film consists of 43 short film sequences that are interrupted by 44 multichoice questions for example: “What is Elin thinking when Daniel is saying …” (cognitive social cognition- inferring thoughts and motives) or “What is Daniel feeling …” (perceptive – seeing and interpreting feeling states). The DMASC-MC text was developed by Dziobek et al. [29] and adapted to Swedish and filmed using bilingual native speaking Swedish actors by Bölte et al. [23]. The exact same test is available in English with native English-speaking actors, hence the term “Double” Movie of Assessment of Social Cognition – Multiple Choice (DMASC-MC). The multiple-choice question appears on the screen for a maximum of 40 s with the participant being asked to say the letter A, B, C or D aloud to the test leader for the answer they think best describes what a particular character’s intention, thoughts or feelings are. The maximum score is 44 for total social cognition (score of 1 for each answer indicating appropriate “social cognition”), 20 for perceptive social cognition and 24 for cognitive social cognition. The scale also allows ratings for concrete thinking, overinterpreting social cognition (hypermentalising) and hypomentalising (where one under-interprets the situation but without using concrete patterns of answering the question in DMASC-MC. These are not examined in this study. The test administrator records the answer on a paper scoring sheet hidden from view of the participant. The movie was presented from a DVD on a computer screen with good quality speakers alongside the computer. The participant can click to the next video segment when ready. If the participant had trouble reading Swedish whilst having a reasonable command of verbal language skills, the instructor read in a neutral voice the possible answers to the participant at the appropriate time. Coding was done according to the protocols of the DMASC-MC [23]. Total time for the test varied between 30 and 45 min.

To assess general cognitive abilities, participants in the SFPP were administered the matrix reasoning and information subtests of Wechsler Adult Intelligence Scales fourth edition (WAIS-IV) by an experienced psychologist or psychologist in training. The choice of these WAIS-IV subtests was based on information and matrix reasoning being robust measures of premorbid intellectual functioning and subtests where scores tend to be consistent between community controls and individuals with schizophrenia [30].

### Ethics

All procedures were in accordance with the Swedish Research Councils ethical guidelines and the Helsinki declaration. Approvals from Stockholm Regional ethics committee were 2014/827–31/4, 2017/ 219–32 and 2018/307–32 for the SFCP participants and 2010/2003–31/3 for normative data acquisition in community controls. All subjects provided written informed consent.

### Statistical analysis

Demographic variables and self-rating scale scores are shown in Table 1. T-tests were used to compare groups on continuous baseline variables, apart from SAPS and antipsychotic dose which were compared using Mann-Whitney Statistic. Chi-square was used when comparing groups for frequencies. Spearman rank correlations were used to ascertain correlations with SAPS, antipsychotic doses and DMASC-MC scores. The correlational analyses were done in order to ascertain if indeed our study could confirm previous findings of correlations between cognitive ability and social cognition scores.

**Table 1:** Demographic data

	Psychotically driven aggression *n* = 49 (range or percentage)	Other aggression *n* = 31 (range or percentage)	Community controls *n* = 81 (range or percentage)
**Sex** – males	38 males (77.6)	24 males (77)	60 (74.1)
**Age**	36.85 ± 11.3 (20–61)	34.6 ± 9.8 (20–57)	37.9 ± 11.5 (21–59)
**Education completed**			
- primary school	24 (32.6)	20 (64.5)	5 (6.2)
- secondary school	19 (38.7)	9 (29.0)	41 (50.6)
- vocational or university training	8 (16.3)	2 (6.5)	35 (43.2)†
**Swedish Schooling before age 15**	38 (77.5)	25 (80)	30 (93.7) ^a^
**History of Substance Abuse**			
-none	9 (24.5)	7 (22)	28 (87.5)^a^
- cannabis	12 (24.8)	2 (6.4)	1 (3.1)^a^
- opiates	2 (4.0)	0	0
- stimulants	0	1 (3.2)	0
- alcohol	6 (12.2)	2 (6.4)	0
- polysubstance abuse	19 (38.8)	19 (61.3) *	3 (9.4) ^a^ ††
**Diagnosis**			
- Schizophrenia/schizoaffective disorder	42 (85.7)	22 (71)	
- Bipolar disorder - psychotic	2 (4.1)	4 (12.9)	
- Delusional disorder and other psychotic disorders (not drug induced)	5 (10.2)	5 (16.1)	
**Duration of Illness** (years)	10.1 ± 7.6 (1–31)	8.9 ± 7.0 (0.5–31)	
**WAIS**			
- information	8.1 ± 3.3 (3–14)	9.0 ± 3.5 (3–15)	10.0 ± 2.36 (6–15) ^a, #^
- matrix reasoning	7.3 ± 3.2 (2–16)	7.9 ± 3.6 (3–14)	10.6 ± 2.83 (3–16) ^a, ##^
**Earlier autism spectrum disorder**	5 (10.2)	7 (22.6)	0 ^a^
**Antipsychotics**			
- typical	20 (40.8)	13 (54.1)	0 ^a^
- atypical	10 (20.4)	9 (37.5)	
- clozapine and depot injection	4 (8.2)	1 (4.2)	
- combinations of typical and atypical	15 (30.6)	1 (4.2) *	
**Antidepressants**	6 (12.2)	6 (25)	0 ^a^
**Mood stabilizers**			
- lithium	1 (2.0)	2 (8.3)	
- others	8 (16.3)	3 (12.5)	
**Haloperidol equivalent dose** (mg/day)^b^	15 (0–70)	6.5 (2–25) **	0 ^a^
**SANS**	24.0 ± 11.3 (2–50)	25.4 ± 16.1 (4–60)	3.37 ± 5.16 (019) ^a^
**SAPS** ^**b**^	3 (0–36)	3.0 (0–53)	0 (0–11) ^a^
**Type of aggressive acts** –			
- threats	11 (22.4)	3 (9.7)	
- assaults	22 (44.9)	20 (64.5)	
- assault with weapons	14 (28.6)	7 (22.6)	
- manslaughter/murder	2 (4.1)	1 (3.2)	
**Number of assaults/threats**			
none known	0	0	0 ^a^
1–2	12 (24.5)	4 (12.9)	
3–5	14 (28.6)	10 (32.3)	
6–9	12 (24.5)	7 (22.6)	
> 10	11 (22.4)	10 (32.2)	

^a^ Based on the 32 controls that have data on these variables. ^b^ Median. † education χ^2^ = 50.23, *p* < 0.00001, †† substance use none, THC, polysubstance use χ2 = 43.56, *p* < 0.00001, # T-test PDA vs healthy controls − 2.83 df 76, *p* = 0.006, ## T-test PDA vs healthy controls − 4.72, df 76, *p* < 0.0001, T-test non PDA vs healthy controls =3.28, df 58, *p* < 0.002 * χ^2^ = 7.96, *p* = 0.047, ** MW-U test, Z-3.58, *p* = 0.00034

For analysing DMASC-MC scores between all three groups non-parametric Kruskal-Wallis rank test was used. Differences between the two PSD groups and individually against controls were calculated using Mann-Whitney U-test. Bonferroni corrections were thereafter used to correct alpha level for multiple comparisons. Analysing DMASC-MC results we found two individuals in the community control group who had statistically extreme values of 12 and 15 out of 44 for total social cognition score on DMASC-MC. We present the DMASC-MC results including these two individuals as uncorrected and as corrected (when we remove these two individuals from the analyses) in Table 2. Multiple regression analyses were used to elucidate effects of relevant variables including social cognition scores on PDA/non/PDA/no aggression using imputation of the mean where there were missing values. A post hoc general linear method was used to examine interactive effects of the variables on PDA/non-PDA/no aggression and Mann Whitney was used to examine differences between the SFPP control group and that obtained from the validation study of DMASC-MC. All statistics were performed in Statistica 13.2.

**Table 2:** Mann Whitney analyses of DMASC-MC differences across groups

Group and Z adjusted, *p*-value	DMASC social cognition		DMASC perceptive		DMASC cognitive	
Non-PDA versus PDA	3.37	*p* < 0.0008^a^	3.19	*p* < 0.0015^a^	2.48	*p* < 0.0133
PDA versus controls (Uncorrected)	−7.55	*p* < 0.0001^a^	−6.09	*p* < 0.0001^a^	−6.80	*p* < 0.0001^a^
PDA versus controls (Corrected)	−7.88	*p* < 0.0001^a^	−6.42	*p* < 0.0001^a^	−7.05	*p* < 0.0001^a^
Non-PDA versus controls (Uncorrected)	−3.24	*p* < 0.0012^a^	−1.91	*p* = 0.0555	−3.15	*p* = 0.0016^a^
Non-PDA versus Controls (Corrected)	−3.49	*p* < 0.0005^a^	−2.15	*p* < 0.0312	−3.37	*p* < 0.0008^a^

Kruskal Wallis for all three groups Social cognition H (2, *n* = 159) = 64.25, *p* < 0.0001, Perceptive H (2, *n* = 159) = 41.68, *p* < 0.0001, cognitive H (2, *n* = 159) = 51.08, *p* < 0.0001. Corrected values obtained after excluding 2 extreme values in the community control group ^a^significant after Bonferroni correction for 15 comparisons

## Results

### Demographic and clinical characteristics

Results are shown in Table 1. The PSD sample, of which 77.5% were male, was relatively poorly educated with 55% not having completed or passed more than 9 years of primary schooling. This was a significant difference between all community controls and PSD groups but not between PDA and non-PDA groups. Whilst the vast majority had Swedish education before age 15, 36.2% were born in countries outside of the Nordic countries (10% in Europe, 11.2% in Middle East, 8.8% in Africa and 6.2% in other world regions). The majority had grandparents born outside of Sweden (57.5%). 80% had a DSM-5 diagnosis of schizophrenia or schizoaffective disorder and were treated with depot antipsychotics of either typical or atypical form. Eleven had a diagnosis of mild intellectual disability, 8 in the PDA group 3 in the non-PDA group (χ^2,^ Yates corrected ns). Of the patient sample, 61.25% had shown aggressive acts in direct response to hallucinations or delusions. 80% had committed more than 3 aggressive acts in their lives and 26.25% had committed 10 or more documented aggressive acts. Polysubstance use was more common in the non-PDA (conflict in relationships or instrumental aggression), than in the PDA group (having acted on their hallucinations or delusions) (61.3% vs 38.8% χ^2^ = 3.86, *p* = 0.0495). The PDA group received higher doses of antipsychotic medications. Both PSD groups had mean age scaled scores on the WAIS-IV subtests in the lower end of the normal range (information PDA mean 8.1, non-PDA 9.0) or in the weak intellectual range (matrix reasoning, mean 7.3 PDA, 7.9 non-PDA) compared with normal range intellectual functioning in the community controls. Information score was statistically lower in the PDA group compared with the community controls whereas matrix reasoning was impaired in both PSD groups compared with healthy controls (see Table 1). There were no significant differences between the PSD groups.

### Social cognition

**Fig. 1:**
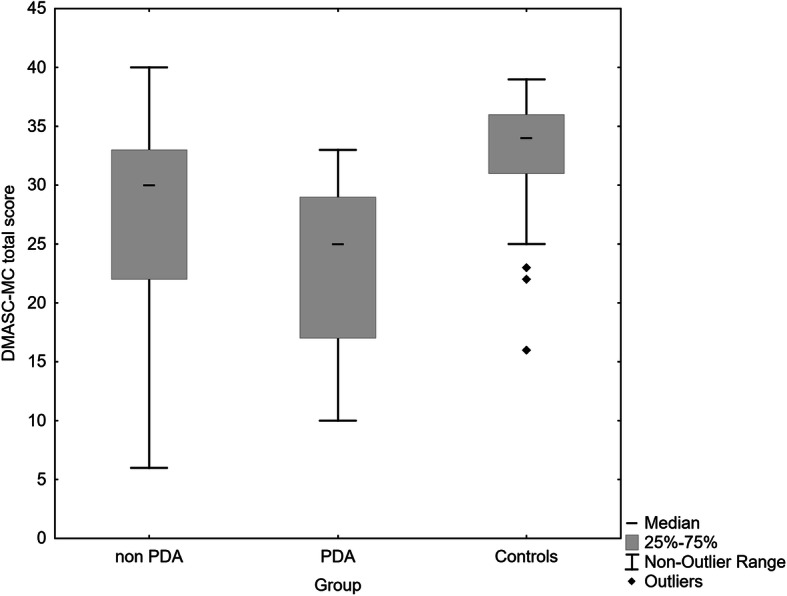
Total social cognition score, Median scores non-PDA 30, PDA 25, healthy controls 34

**Fig. 2:**
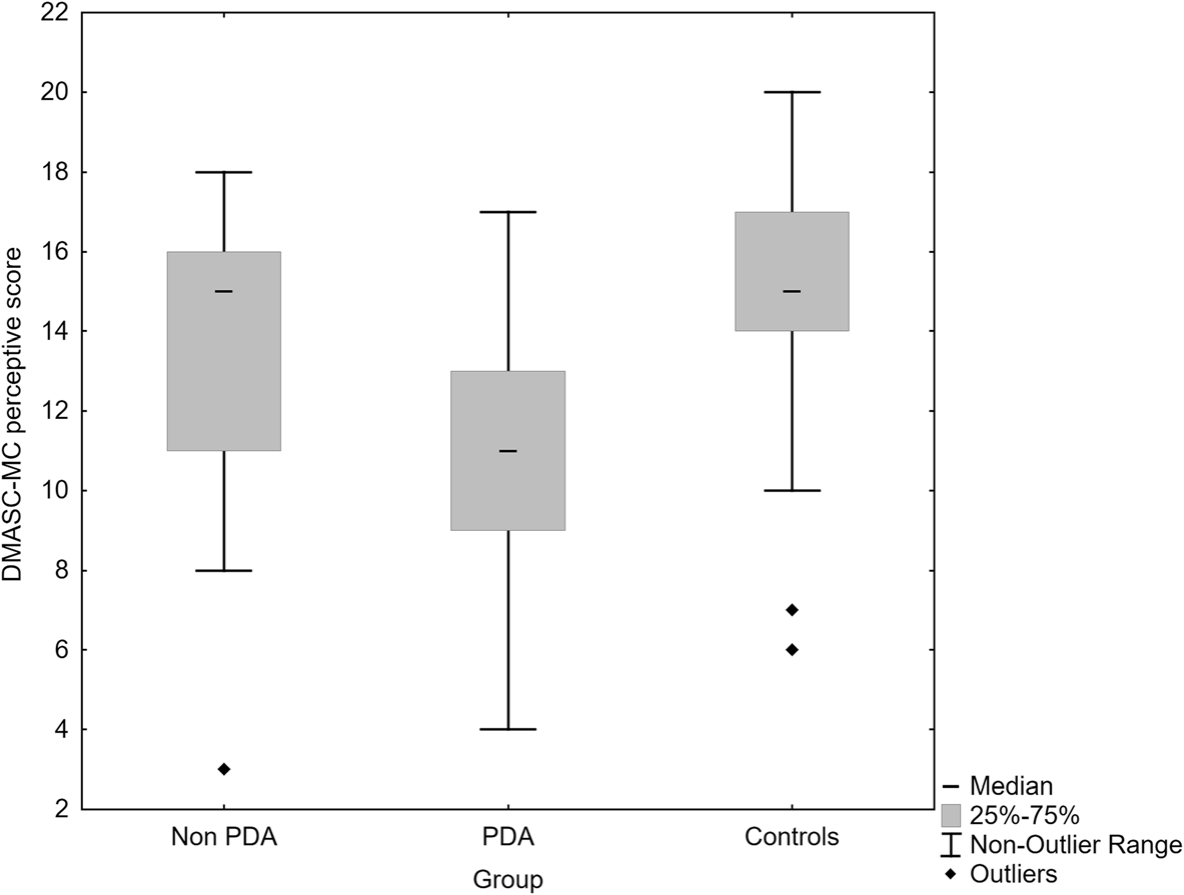
Total perceptive social cognition score, Median scores non-PDA 15, PDA 11 healthy controls 15

**Fig. 3:**
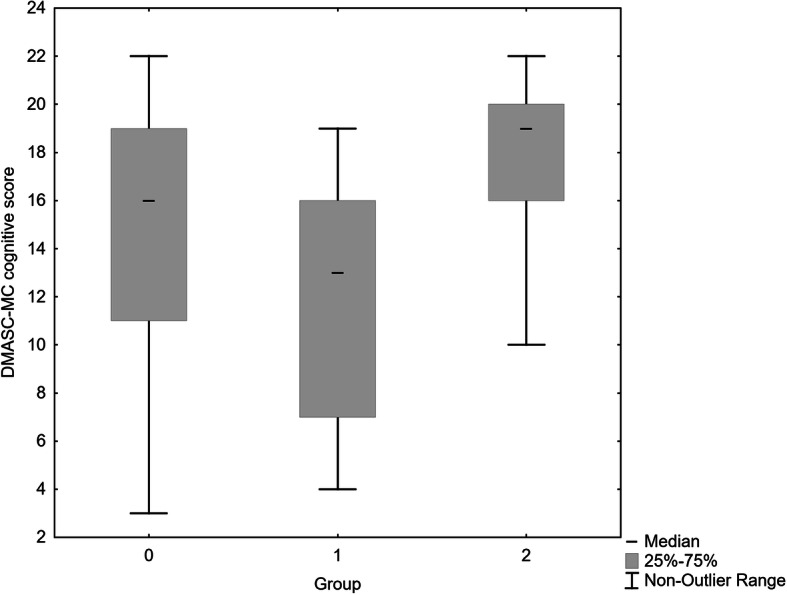
Total cognitive social cognition score. Median scores non-PDA 16, PDA 13, healthy controls 19

Figures 1, 2, 3 show the median and distribution of scores with 25–75 percentiles in all three scales of the DMASC-MC. In short, the PDA group was most impaired on all measures, those with non-PDA had similar perceptive scores to community controls (median score both 15, compared with 11), whereas they were intermediate on cognitive social cognition (median score 16, compared with 13 PDA, and 19 community controls). As can be seen there was, however, overlap between the three groups in their scores. Kruskal Wallis test yielded statistical differences in DMASC-MC scores: social cognition H (2, *n* = 159) = 64.25, *p* < 0.0001; perceptive H (2, *n* = 159) = 41.68, *p* < 0.0001; cognitive H (2, *n* = 159) = 51.08, *p* < 0.0001, Table 2. Even after Bonferroni correction of Mann Whitney comparisons (*p* < 0.0033), the PSD group with a history of PDA exhibited lower total and perceptive social cognition than the non-PDA group (and were lower on these as well as on cognitive social cognition compared with controls), see Table 2.The non-PDA PSD group scored lower than community controls on total and cognitive social cognition but not on perceptive social cognition.

### Correlations with social cognition

Uncorrected correlational analyses were used as a tool to ascertain which variables were important to consider when examining whether aggression type was independently associated with social cognition ability. Table 3 shows Spearman rank order correlations of potentially significant clinical variables that may confound our results. As can be seen, the correct understanding of emotion synonyms was highly correlated with all three measures of social cognition but after performing a Bonferroni correction significance remained only for total and cognitive social cognition scores. WAIS-IV subscale scores were also positively correlated with all the DMASC-MC scores. In only examining the PSD group there were expected but very weak negative correlations with dose and symptomatology on social cognition. None of these correlations were significant.

**Table 3:** Spearman rank order correlations of variables with DMASC scores

	Total Social cognition score		Perceptive social cognition score		Cognitive social cognition score	
Synonym words	0.34	< 0.001*	0.28	< 0.01	0.34	< 0.001*
WAIS matrix	0.59	< 0.00001*	0.42	< 0.00001*	0.62	< 0.00001*
WAIS information	0.48	< 0.00001*	0.38	< 0.0001*	0.49	< 0.00001*
Haloperidol dose/ day ^a^	−0.23	0.04	− 0.22	ns	−0.19	ns
SANS ^a^	−0.13	ns	−0.14	ns	−0.11	ns
SAPS ^a^	−0.11	ns	−0.15	ns	−0.04	ns

^a^ Based only individuals with PSD. * Remains significant after Bonferroni correction (*p* < 0.0028)

### General intellectual ability, type of aggression and social cognition

The identified factors in the correlational analyses (WAIS-IV matrix and information scores as well as synonym score) along with symptom scores and haloperidol dose were used in a multiple regression along with total social cognition scores. Table 4 shows that total mentalizing scores did predict prior aggression when considering all three groups. There were no effects of WAIS-IV subtest scores, synonym understanding or of positive symptoms on the type of aggression exhibited. Current Haloperidol dose equivalence and negative symptoms did predict prior aggression but can be understood as reflecting the patient/healthy control divide in the analysis of all three groups.

**Table 4:** Multiple regression of mentalising on PDA

	B	*p*-value	Adj R^2^ *p*-value
PDA all three groups ^a^			
WAIS Matrix	0.02	ns	
WAIS Information	−0.03	ns	0.33
Synonyms	0.005	ns	*p* < 0.0001
DMASC-MC total	0.018	0.047	
Haloperidol mg/d	−0.020	0.0006	
SANS	−0.018	0.0002	
SAPS	0.002	ns	
PDA total PSD groups ^b^			
WAIS Matrix	0.017	ns	0.22 *p* < 0.0001
WAIS information	−0.002	ns	
Synonyms	0.016	ns	
DMASC-MC total	−0.026	0.0011	
Haloperidol	0.018	0.0003	
mg/day	−0.005	ns	
SANS	−0.009	ns	
SAPS			

^a^F (4,151)=10.76, ^b^F (4,72)=4.16

Social cognition became an even stronger determinant of PDA when just considering the PSD groups. Unsurprisingly, there were no effects of current SAPS or SANS scores on past aggression type yet greater haloperidol dosages at the time of DMASC-MC testing was a predictor of PDA. This can be interpreted to mean that the PDA group was judged initially to have had either greater positive symptom burden or been more difficult to treat such that dosages in this group needed to be larger in order to alleviate symptoms. There were no effects of cognitive ability or synonym understanding on PDA.

### Further analyses

Table 5 shows a post hoc general linear regression analysis analysing the effect of social cognition scores on past aggression form when taking into account the interactive effects of current symptomatology and antipsychotic dose as well as the interactive effects of measures of cognitive abilities. As seen, whilst the whole model was significant in explaining differences when all three groups were studied, social cognition scores did not exert an independent effect. However, examining just the PSD groups, social cognition scores were the only predictor of PDA.

**Table 5:** Interactive effects of variables on PDA

		F	*p*-value	Adj R^2^	Whole model *p* value
All three groups	DMASC-MC total score Haloperidol mg/day	1.77	ns	0.06	0.027
	*SANS*SAPS WAIS Matrix*WAIS	0.93	ns		
	Information*synonyms	1.32	ns		
PSD groups	DMASC-MC total score Haloperidol mg/day	6.39	0.014	0.092	0.021
	*SANS*SAPS WAIS Matrix*WAIS	1.60	ns		
	Information*synonyms	0.13	ns		

In order to elucidate whether diagnosis may have impacted the results, Mann Whitney statistic was calculated for total social cognition, cognitive and perceptive social cognition of bipolar disorder versus schizophrenia and of other psychoses compared with schizophrenia. There were no significant differences in social cognition scores (Z adj ranging from 0.09 to 0.81, p ranging from 0.42 to 0.92). Given the possible confounder of a history of substance abuse on social cognition, we re-ran the regression analysis removing those participants with a history of substance abuse. The results remained significant at the 3-group level for matrix score (b = 0.99, *p* < 0.001) and the type of aggression (b = 1.20, *p* = 0.042), F (4,42) = 4.50, *p* < 0.0001. There were too few non-substance abusers in the PSD groups (*n* = 16) in order to get a meaningful result examining just the PSD groups.

To ascertain if there were differences in social cognition between those community controls specifically recruited for SPFF and those from the validation study we performed post-hoc Mann-Whitney analyses just comparing these 2 groups revealing no differences on social cognition Z adj = 0.306, *p* = 0.76, perceptive social cognition Z adj = 1.498, *p* = 0.134, cognitive social cognition Z adj = 1.578, *p* = 0.115.

Posthoc power analysis revealed power of > 0.99 for total social cognition as a function of PDA/non PDA/non-aggression in one way ANOVA and multiple R^2^.

## Discussion

In this study of previously aggressive, stable and medicated individuals with PSD, all of whom had abstained from illicit substances and alcohol for at least 3 months, we found lower social cognition abilities as measured by DMASC-MC in those who had committed aggressive acts because of internal stimuli in the form of delusions or hallucinations (PDA) than in 1) those who had shown interpersonal or instrumental aggression (non-PDA) as well as 2) community controls. The PSD group with a history of PDA exhibited lower perceptive social cognition but not cognitive social cognition compared with the non-PDA group and performed inferior on both these measures compared with community controls. The non-PDA PSD group scored lower than community controls on overall and cognitive social cognition but not on perceptive social cognition. In examining social cognition as a predictor of past PDA we found that social cognition, but not cognitive ability was a significant predictor. In our study the PDA group had similar levels of current positive symptoms to the non-PDA group but had been treated with higher doses of antipsychotic medications, perhaps reflecting a more severe form of illness.

Whilst both PSD groups were impaired on social cognition ability, lower general cognitive abilities, as operationalized by general knowledge and nonverbal abstract logical reasoning on the WAIS-IV, were powerful predictors of impaired social cognition ability in the PSD groups. This finding is in line with other studies of schizophrenia (see review by Bora and Pantelis, [5]), yet the finding as it relates to intellectual disability, which is present in a proportion of our PSD individuals is less clear as there is a dearth of studies examining this possible link in a non-autistisc group. However, despite the effects of cognitive abilities on social cognition, a history of psychotically driven aggression remained a significant predictor of lower social cognition, even after adjustment for differences in general, non-social, cognitive abilities.

To the best of our knowledge, this is the first study of PSD to examine differences in social cognition between those whose aggression has been driven by psychotic symptoms and those with other forms of aggressive acts in PSD. The distinction of the nature of aggressive acts in those with psychoses remains important in many jurisdictions around the world as to the legal consequences incurred by that individual, especially in cases of severe assaults or murder. Here we suggest that impairment in social cognition ability may be a trait marker which predisposes a person with PSD to misinterpreting interpersonal cues in the environment. Possibly traits of impaired perceptive social cognition are further detrimentally affected by psychotic symptoms during acute episodes, rendering them unable to generate alternative hypotheses to situations and becoming more prone to paranoid interpretations which in turn predispose them to acting on their delusions and hallucinations.

That we found greater impairment in social cognition ability in those with PDA could suggest that these were more severely affected by positive symptoms, but we found no support for symptoms impacting on social cognition scores in the present. However, we did find higher dosages of antipsychotic dosages being used at the time of testing with DMASC-MC achieving the same level of positive symptom burden at a group level. This could either mean that that this group has had more positive symptoms at the time of the aggression (more severely ill) and needing higher doses to treat or is less sensitive to antipsychotic medications. Another hypothesis would be that incorrect social cognition conclusions are interpreted as delusions, regarded potentially as more severe psychotic symptoms that are to be treated more aggressively. Possible links between social cognition and persecutory delusions could not be tested in the study as all bar five PSD participants had experienced these, which in itself is interesting and awaits further analysis when a non-aggressive patient sample has been fully recruited.

Several studies have examined social cognition in schizophrenia and aggression. No differences were found in faux pas tests of cognitive social cognition [19, 31] or in an empathic accuracy test [31] between those with schizophrenia with and without aggression. However, the studies did not differentiate different forms of aggression nor include cognitive measures. The study by Majorek et al. [31] showed that patient groups with schizophrenia were more impaired on a theory of mind test than unaffected controls, yet those who had been aggressive performed poorer than the non-aggressive group on questions regarding the test but were similar in sequencing the pictures in the stories. Again, there was no attempt to differentiate between different aggression motives. The study by O’Reilly et al. [18], whilst prospective in terms of identifying probands with new episodes of aggression during extended forensic inpatient stays, did not differentiate between previous PDA and other types of aggression. They identified that recidivists in aggression during the follow-up period were more impaired in both neurocognition and the Mayer-Salovey-Caruso Emotional Intelligence Test (MSCEIT). Yet, the aggressive recidivist group was small (*n* = 10) compared with the comparison group (*n* = 79) and little detail was provided regarding offending history. It is also uncertain how aggression on long stay inpatient wards correlate with aggressive acts in other environments. The study by Bo et al. [14] used a rater-based adapted instrument “Metacognitive Assessment Scale” (utilizing videotaped segments of a PCL-R interview – Hare Psychopathy Checklist-revised) and found that higher psychopathy scores and lower emotional awareness of other’s states correlated with instrumental aggression. Psychotically driven aggression was not rated. Engelstad et al. [17] showed large impairments in social cognition on the Norwegian adaptation of the MASC in homicide offenders with schizophrenia compared with nonviolent persons with schizophrenia and community controls.

That our PSD sample was impaired on social cognition ability compared with normative controls is consistent with previous research using the DMASC-MC or MASC. Our PSD participants who had not committed PDA were similar on the DMASC-MC to non-aggressive schizophrenia individuals in the studies by [6, 7, 17]. In our study, we had no knowledge of psychiatric or neurodevelopmental conditions in a proportion of community controls which may explain the outliers and extreme values we saw in a couple of individuals. The individuals with PDA had DMASC-MC scores on par with the schizophrenia group found by Montag et al. [32] and for those who had not committed homicide in Engelstad et al.’s study [17]. A possible explanation of the lower social cognition scores in our group is the inclusion of individuals with mild intellectual disability, who usually have been excluded by previous research. Other studies mostly included individuals whose mean IQ ranged between 90 and 100. The decision to include mild intellectual disability was based on ecological validity and generalizability of findings to the group of persons who have double and triple diagnoses, who make up a disproportionate number of those who commit aggressive acts. The issue of including persons with past substance abuse is, of course, an added complication in terms of explaining causality of impairments, as polysubstance use has been shown to correlate with impaired emotional social cognition [33], at least during active use. It is unknown if these impairments existed prior to drug abuse or if they remit during prolonged abstention, which were the circumstances of our PSD participants. While we had insufficient numbers of persons who had not abused substances in our PSD groups to just analyse these, when combining the three groups we still found an effect of aggression/type of aggression on social cognition scores. Yet, obviously this could be more related to aggression per se than psychotically driven aggression.

Although there is a burgeoning corpus of studies regarding the probable positive effects of training social cognition in schizophrenia [34] there is a smaller but emerging literature suggesting that social cognition training, especially in conjunction with cognitive remediation, can reduce aggression recidivism in individuals with schizophrenia with a history of aggression [35]. If the results of our study are replicated, it would be interesting to know if the PDA and non PDA groups respond similarly to such interventions or if there needs to be tailoring of interventions according to the mechanisms underlying the aggressive acts.

A strength of our study is the inclusion of participants who have comorbidities in terms of previous substance abuse and mild intellectual disability creating more ecologically valid and generalizable findings to the group of persons who commit aggressive acts, mostly during psychotic episodes. Additionally, variation in diagnosis or symptom ratings were minimized by having only two raters of aggression and two raters of symptom and diagnoses utilizing a variety of documented sources. Using a measure of social cognition that has been used in many different research studies of schizophrenia and autism spectrum disorders, the DMASC-MC, yields directly comparable results with other studies.

There are several limitations of the study. Firstly, the use of two separate groups of community controls, where not all the study parameters are known in the group not specifically recruited for SFPP, and where the multiple regression analyses are based on imputation of the mean in those persons where data are not known. This means that the results must be viewed with some caution and the study will be validated when there are sufficient community controls recruited within SFPP. Secondly, the enrolment of individuals with varied diagnoses and histories of intellectual abilities as well as prior substance use mean we cannot extrapolate our results to specific diagnoses. Rather the study examines the PSD group of individuals who have shown aggression, likely because of several different factors, but where little is known about commonalities in the group, which this study attempts to add knowledge about. The study has a cross-sectional design limiting the interpretation of causality. Another limitation is the use of the DMASC-MC, relying on social cognition in a situation not linked to aggressive acts, and which depicts a social situation individual in the study may not have been personally exposed to. Together with changing interaction patterns with the advent of social networking sites on the internet and problems with abstract thinking in our study group, DMASC-MC may not prove to be a fully valid method of tapping into social cognition, as opposed to abstraction capabilities or having watched sufficient number of films and TV where similar situations are portrayed and “answers given”. Another limitation of the study is the lack of a non-aggressive PSD group to show if any of the impairments are specific to those who have been aggressive or simply reflect severity of the schizophrenia/bipolar deficits. Recruitment of this group is underway along with a larger community control group which we will use to validate the results of this study. Larger studies are therefore needed to confirm or refute our results. Finally, we had little information on the community control group, aside from recruitment, basic demographics and DMASC-MC results, potentially introducing a selection bias.

## Conclusion

Psychotically driven aggression and general intellectual function were the most powerful contributors to social cognition ability in this sample of previously aggressive persons with PSD. Both PSD groups (PDA and non-PDA) showed impairment in social cognition compared with community controls. Yet the group who had committed psychotically driven aggression were most impaired in perceptive social cognition while both PSD groups were impaired on cognitive social cognition, likely linked to the presence of the psychotic spectrum disorders per se. Current antipsychotic dose and social cognition ability predicted past history of PDA in the PSD groups. Future studies need to compare social cognition in these groups with a non-aggressive PSD group in order to elucidate whether social cognition is coupled to the form of aggression per se or is disease severity related.

## Data Availability

Data will be made available on request by contacting anette.johansson.2@ki.se.

## Abbreviations

ASPD: Antisocial personality disorder
DMASC-MC: Double movie for the assessment of social cognition – multiple choice
DSM-5: Diagnostic and statistical manual-5
DVD: Digital versatile disc
KIND: Centre for Neurodevelopmental disorders at Karolinska Institutet
PCL-R: Hare psychopathy checklist – revised
PDA: Psychotically driven aggression
SANS: Scale for the assessment of negative symptoms
SAPS: Scale for the assessment of positive symptoms
SCAN2.1: Schedule for clinical assessment in neuropsychiatry
SFPP: Stockholm forensic psychiatry project
PSD: Psychotic spectrum disorders
WAIS-IV: Weschler adult intelligence scale-4th edition
